# Pharmacokinetics of phenoxodiol, a novel isoflavone, following intravenous administration to patients with advanced cancer

**DOI:** 10.1186/1472-6904-11-1

**Published:** 2011-02-03

**Authors:** Jan B Howes, Paul L de Souza, Leanne West, Li Jiu Huang, Laurence G Howes

**Affiliations:** 1 Department of Pharmacology and Therapeutics, Griffith University, Gold Coast Hospital, Southport, Queensland, 4215 Australia; 2 Cancer Pharmacology and Therapeutics Lab, St. George Hospital Clinical School, Kogarah, NSW, 2217 and University of NSW, Australia; 3 Marshall - Edwards Pty Ltd, 140 Wicks Rd. North Ryde, NSW, Australia; 4 University of Western Sydney Medical School, Campbelltown, NSW 2560, Australia

## Abstract

**Background:**

Phenoxodiol is a novel isoflavone currently being studied in clinical trials for the treatment of cancer. This study reports the pharmacokinetics of phenoxodiol in patients with cancer.

**Methods:**

The pharmacokinetics of phenoxodiol was studied following a single intravenous (iv) bolus dose and during a continuous intravenous infusion. Three men with prostate cancer and 3 women with breast cancer received IV bolus phenoxodiol (5 mg/kg) and plasma was sampled for free and total phenoxodiol levels. On a separate occasion 5 of the same patients received a continuous intravenous infusion of phenoxodiol (2 mg/kg/h) and plasma was again sampled for free and total phenoxodiol levels. Phenoxodiol was measured using gradient HPLC with ultraviolet detection.

**Results:**

Following bolus injection, free and total phenoxodiol appeared to follow first order pharmacokinetics. The elimination half-lives for free and total phenoxodiol were 0.67 ± 0.53 h and 3.19 ± 1.93 h, respectively, while the total plasma clearance rates were 2.48 ± 2.33 L/h and 0.15 ± 0.08 L/h, respectively. The respective apparent volumes of distribution were 1.55 ± 0.69 L/kg and 0.64 ± 0.51 L/kg. During continuous intravenous infusion, free phenoxodiol accumulated rapidly to reach a mean concentration at steady state of 0.79 ± 0.14 μg/ml after 0.87 ± 0.18 h. The apparent accumulation half-life of free phenoxodiol was 0.17 ± 0.04 h while the plasma clearance during continuous infusion was 1.29 ± 0.23 L/h.

**Conclusions:**

Phenoxodiol has a short plasma half-life, particularly in the free form, leading to a rapid attainment of steady state levels during continuous intravenous infusion.

**Trial registration:**

Australian New Zealand Clinical Trials Registry (ANZCTR): ACTRN12610000334000

## Background

Phenoxodiol (PXD, NV-06) is an isoflavone derivative that has been demonstrated *in vitro *and *in vivo *to possess anti-cancer activity [1-9]. Phenoxodiol is a synthetic analogue of genistein with unknown mechanism of action. It appears to have pleiotropic actions such as inhibition of tyrosine kinases, inhibition of topoisomerase II in a dose-dependent manner and inhibition of the X-linked inhibitor of apoptosis [2,10-12] but the predominant mode of action remains to be elucidated. A series of intriguing observations were made by Morre et al. [13-16], who showed that phenoxodiol acts by inhibiting ENOX2 (tNOX), a tumour-associated cell surface ubiquitinol (NADH) oxidase, which functions as an alternative pathway for plasma membrane electron transport. Further, phenoxodiol has the ability to modulate chemoresistance and promote apoptosis of ovarian cancer cells [2,17,18]. These anti-cancer properties have generated much interest in phenoxodiol as a potential cancer treatment.

Phase I studies of phenoxodiol have been previously published [19,20], and the molecule was being studied in a Phase III clinical trial in the treatment of ovarian cancer until recently, when the study was halted prior to full accrual. At the time this study was performed in 2000, we had no information regarding the behaviour of phenoxodiol in humans, so the first study undertaken was a pharmacokinetic study to investigate both bolus and continuous intravenous administration. This paper presents data derived from that first-in-human, pharmacokinetic study, and our aim at the time was to determine the pharmacokinetic parameters of phenoxodiol in order to inform us further about the design of Phase I and other studies.

## Methods

A single centre, open-label pharmacokinetic study involving six patients with metastatic cancer was performed between April 2000 and November 2000. Each patient was recruited in a serial manner and given a single intravenous (iv) injection of phenoxodiol over a five minute period at a dose of 5 mg/kg initially. At least 6 weeks after their individual intravenous bolus administration, each patient was then given a continuous infusion of phenoxodiol at a rate of 2 mg/kg per hour for a duration of up to ten times the average half life determined from the data obtained after intravenous bolus dosing. No continuous intravenous infusion occurred until all the pharmacokinetic data from intravenous bolus dosing was available. The intravenous bolus dose of phenoxodiol was selected as 10% of bolus intravenous doses achievable in mice up to 50 mg/kg that had demonstrated no discernible toxicity, including histopathological evaluation (data on file, Novogen Pty Ltd).

### Subjects

Six patients were studied in total. The inclusion criteria were: male or female volunteers with metastatic disease from any solid tumour, age 18 to 70 years, normal haematological parameters, and a prognosis of at least three months. Exclusion criteria were: leukemia or lymphoma, allergy to soy products, vegetarian diet or use of soy product more than three times a week, the use of sex steroids in the previous two months, antibiotic therapy within one month prior to the study period or at any time during the study, smoking of greater than 10 cigarettes/day, and the known presence of central nervous system metastases. Active infection or other co-morbid disease that in the opinion of the investigator would have precluded the patient from participating in the study was also an exclusion criterion. Patients also had to have relatively stable or slow growing tumours and not commence new anti-cancer therapies while on study. The study was approved by the Ethics Committee of St George Hospital (00/13 Howes), and patients had written, fully informed consent.

### Study protocol

Screening procedures included a medical history, full physical examination, haematology and biochemistry. Volunteers were then assessed for compliance with the inclusion and exclusion criteria. All patients were instructed to maintain a diet low in isoflavones for one week prior to and for the duration of the study, and to arrive at the hospital on the treatment day at approximately 0800 having fasted and abstained from caffeine and alcohol for 24 hours (h). An initial blood sample of 40 ml was drawn from an antecubital vein-indwelling catheter at baseline prior to study medication administration. Blood draws were planned at 0.0 (baseline, prior to infusion), 0.5, 1.0, 1.5, 2.0, 2.5, 3.0, 4.0, 5.0, 6.0, 7.0, 8.0, 10.0 and 12.0 h after the administration of phenoxodiol. A standard, light, low fat meal was provided 4 hours after the administration of study medication. After observation for 12 hours, the indwelling intravenous catheter was removed and the patient advised not to consume alcohol over the ensuing 24-hour period.

At least 6 weeks after their intravenous bolus dose, patients were recalled individually for their continuous intravenous infusion. The same diet and preparation was required prior to dosing. Fifteen ml of blood was collected at baseline and at 10 and 20 minutes following the start of infusion of phenoxodiol, then at 20 minute intervals up to five hours after the commencement of the infusion. A standard, light, low fat meal was provided 4 hours after the administration of study medication. Both indwelling venous catheters were removed six hours after commencement of the infusion and the patient was then allowed to leave the hospital following a period of observation.

### Safety assessments

#### Laboratory Safety Testing

The following tests were performed at baseline, 24 h, and 48 h after intravenous bolus dosing, and at baseline, 6 h, 5 7 days and 12 14 days after the start of continuous intravenous infusion: full blood count (FBC) including hemoglobin, red cell count, white cell count and differential, platelets, as well as serum biochemistry including total protein, albumin, aspartate aminotransferase (AST), alanine aminotransferase (ALT), gamma-glutamyl transferase (GGT), alkaline phosphatase (ALKP) and urea and electrolytes.

#### Vital Signs and Other Safety Measurements

The patient's weight, height and blood pressure and heart rate were measured at baseline; blood pressure and heart rate were monitored throughout the study.

### Study medication administration

Phenoxodiol was manufactured by Novogen Laboratories, 140 Wicks Road, North Ryde NSW 2119, Australia, and was prepared as an intravenous injection of 100 mg in a 10 ml vial. Samples of the batches of vials used were tested for sterility and for the presence of pyrogens. For single bolus intravenous injection, phenoxodiol was diluted to a total volume of 50 ml in normal saline. The solution was infused over a five minute period using an infusion pump. For continuous intravenous infusion, phenoxodiol was diluted to 500 ml in normal saline and infused using a pump at a constant rate of 100 ml per hour.

### Assay of phenoxodiol levels

Total phenoxodiol levels were measured in a 300 μL aliquot of plasma or urine which was mixed with 15 μL glucuronidase and incubated for 24 h at 24°C prior to extraction. Free phenoxodiol levels were measured in a 300 μL aliquot of plasma or urine which was extracted without prior incubation with glucuronidase. After adding 10 μL of 2, 4, 4'- trihydroxydeoxy-benzoin (0.22 mg/mL) as an internal standard, the sample was extracted using an ethylacetate : hexane (6:4) solution 500 μL. The sample-solvent mixture was centrifuged at 1200 g for 15 min and supernatant was collected and dried under vacuum. The extract was reconstituted in 100 μL 50% isopropanol in water. 5 uL of the extracted sample was injected into the HPLC system. Standards of phenoxodiol were prepared in blank plasma or urine.

HPLC separation was carried out using an Alltech Alltima C18, 5 μm, 250 mm × 2.1 mm column in a 40°C oven. The gradient elution was performed at a flow rate of 0.2 mL/min from mobile phase A (25% acetonitrile, 74.95% water, 0.05% trifluoroacetic acid) to mobile phase B (99.95% acetonitrile, 0.05% trifluoroacetic acid). The fraction of mobile phase A to mobile phase B at 0, 4.5, 9, 11, 12 and 14 minutes were 75:25, 40:60, 30:70, 0:100, 0:100 and 75:25 respectively. With 6 minutes post run, the total run time was 20 minutes. Phenoxodiol and the internal standard (benzoin) were detected by ultraviolet diode array detector at wavelength 335 nm.

The approximate retention time for benzoin and phenoxodiol were 6.7 minutes and 8.4 minutes, respectively. The calibration curve for the assay was linear in the range of 0.25 ug/mL to 10 ug/mL with a correlation coefficient (R^2^)>0.994. The lower limit of quantitation (LOQ) for phenoxodiol was 0.25 ug/mL. Samples with high concentrations were diluted with the same blank matrix before being analysed. As phenoxodiol has very good absorption at 335 nm and blank biological matrix has much less UV absorption at this wavelength, co-elution did not cause any problems with regards to interpretation. Some samples were analysed using both HPLC-UV and LC-MS-MS methods and the results were similar, thus validating our study data.

### Pharmacokinetic analysis

The concentration at steady state (C_ss_) and the time to reach steady state were taken as the actual values when the measured plasma phenoxodiol concentration appeared to reach a maximum. The terminal elimination rate constant (K_el_) was estimated using linear regression analysis using the final plasma sampling times over which the log of the plasma concentration versus time curve appeared to be straight. At least four plasma concentration time points were used for each estimation. The elimination half life (T1/2) was calculated as 0.693/K_el_. The area under the plasma concentration versus time curve (AUC) was calculated using the trapezoid rule with the terminal phase (last measured plasma concentration time point to infinity) calculated as the final plasma concentration divided by K_el_. The apparent volume of distribution (Vd) following acute administration in Part 1 of the study was calculated by extrapolating the log plasma concentration versus time line back to zero time to estimate the theoretical plasma concentration at zero time (C_0_) and dividing C_0 _by the dose. Clearance following acute administration was calculated as Vd multiplied by K_el_.

Accumulation half-lives during continuous intravenous infusions of phenoxodiol were calculated as the time to achieve steady state plasma concentrations divided by 5. Clearance (Cl) was calculated as the infusion rate of phenoxodiol divided by the plasma concentration at steady state (C_ss_). Pharmacokinetic analysis was performed with the assistance of the Excel software plug-in program pkf (Joel I. Usansky, Ph.D., Atul Desai, M.S. and Diane Tang-Liu, Ph.D., Department of Pharmacokinetics and Drug Metabolism, Allergan, Irvine, CA 92606, U.S.A). The results are expressed as the mean values for the subject group along with standard deviations (SD) and ranges.

## Results

### Patients

Six patients (3 males, 3 females) were given intravenous bolus doses of phenoxodiol, but only five went on to receive continuous intravenous infusion. The patient who missed out on continuous intravenous infusion had developed progression of her disease and was advised by her oncologist to proceed with other treatments; there was no evidence that the single administration of phenoxodiol had contributed to her disease progression. Continuous intravenous infusion occurred an average of 56 ± 21 days (range 41-98 days) following intravenous bolus dosing of phenoxodiol. The mean age of the patients was 61.2 ± 9.4 years, their mean weight was 75.3 ± 8.48 kg and their mean height was 169.2 ± 10.0 cm. One patient smoked (5 cigarettes per day), 3 patients consumed between 10 and 30 g of alcohol per day and 3 patients did not regularly consume alcohol.

The 3 female patients had metastatic breast cancer and had a diagnosis of the primary malignancy 15, 7 and one year prior to the study. The 3 males had metastatic prostate cancer and two of these patients had a diagnosis of the primary malignancy 6 years prior to the study. The date of diagnosis of the primary malignancy for the remaining male was unknown. Patients predominately had bone metastases, although one male had known hepatic metastases. One male patient also had a history of laryngectomy for malignant disease. One patient had bilateral leg oedema and hepatic enlargement on physical examination. Clinical examination findings in the remaining patients were unremarkable.

One patient had mild thrombocytopenia at baseline, but haematology parameters were otherwise within normal limits for the remaining patients. Three patients had abnormal liver function tests at baseline; two had serum ALKP levels 3-5 times the upper limit of normal (ULN), one had GGT levels 9 × ULN, and one had a GGT level 1-2 × ULN. All six patients had normal renal function and normal serum electrolyte levels.

### Pharmacokinetics

**Figure 1:**
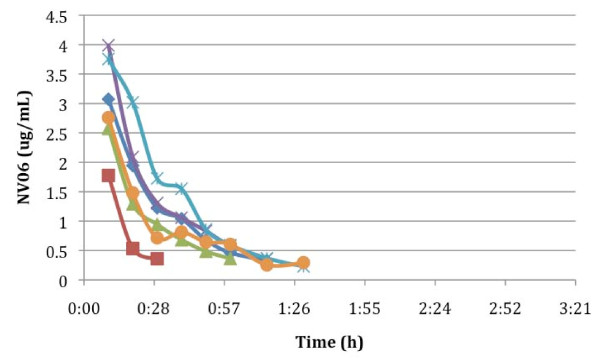
**Free phenoxodiol levels following bolus intravenous administration of 5 mg/kg of phenoxodiol over 5 minutes**.

**Figure 2:**
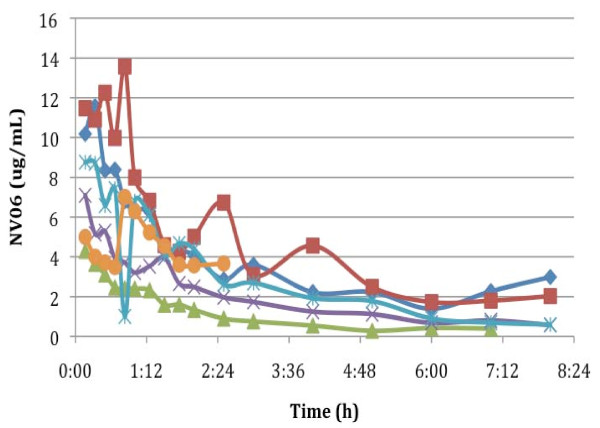
**Total phenoxodiol levels following the bolus intravenous administration of 5 mg/kg of phenoxodiol over 5 minutes**.

Summaries of the pharmacokinetic data for free and total phenoxodiol following the single intravenous bolus dose are presented in Tables 1 and 2. Plasma concentration versus time plots for free and total phenoxodiol for these patients are presented in Figures 1 and 2. Linear regression of log plasma concentration versus time plots indicate that phenoxodiol appeared to follow first order kinetics in all patients (that is, a single phase of elimination from plasma). This was the case for both free and total phenoxodiol. Total phenoxodiol had a longer plasma elimination half-life than free phenoxodiol, but both were relatively short (mean values 3.19 ± 1.93 h and 0.67 ± 0.53 h, respectively). These short half-lives were associated with relatively high mean total plasma clearance rates of 2.48 ± 2.33 L/h and 0.15 ± 0.08 L/h respectively. The apparent volumes of distribution (V_d_) at steady state for free and total phenoxodiol were relatively low (1.55 ± 0.69 L/kg and 0.64 ± 0.51 L/kg, for free and total phenoxodiol, respectively) and total phenoxodiol had a lower volume of distribution than free phenoxodiol. The area under the plasma concentration versus time curves for (AUC) total phenoxodiol following the bolus intravenous dose in Part 1 of the study was approximately 15 times greater than for free phenoxodiol.

**Table 1:** Pharmacokinetic parameters for free phenoxodiol following a single bolus dose of 5 mg/kg infused intravenously over 5 minutes.

Patient	Kel	T1/2 (h)	AUC_0-t _(μg.h/ml)	AUC_0-inf _(μg.h/ml)	Cl (L/h)	Vd (L/kg)
1	1.25	0.55	1.49	1.55 (4.1%)*	1.50	1.20

2	2.42	0.28	0.40	0.40 (1.2%)	7.01	2.90

3	1.59	0.43	1.04	1.05 (13.2%)	2.22	1.40

4	1.24	0.56	1.65	1.66 (10.3)	1.24	1.00

5	0.39	1.74	2.59	2.59 (11.3)	0.46	1.17

6	1.54	0.45	1.21	1.22 (11.8%)	2.46	1.60

Mean ± SD	1.41 ± 0.66	0.67 ± 0.53	1.40 ± 0.73	1.41 ± 0.73 (8.7%)	2.48 ± 2.33	1.55 ± 0.69

Kel is the elimination rate constant. T1/2 is the terminal half life. AUC_0-t _is the area under the plasma concentration versus time curve measured to the last plasma sample. AUC_0-inf _is the area under the plasma concentration versus time curve extrapolated to infinity. Cl is the plasma clearance rate. Vd is the apparent volume of distribution. *The figures in parentheses are the estimated free phenoxodiol proportion for each patient, based on the calculation free AUC_0-inf _x100/total AUC_0-inf_

**Table 2:** Pharmacokinetic parameters for total phenoxodiol following a single bolus dose of 5 mg/kg infused intravenously over 5 minutes.

Patient	Kel	T1/2 (h)	AUC_0-t _(μg.h/ml)	AUC_0-inf _(μg.h/ml)	Cl (L/h)	Vd (L/kg)
1	0.21	3.33	27.10	37.76	0.06	0.31

2	0.25	2.71	33.47	34.64	0.05	0.21

3	0.37	1.85	7.45	7.93	0.29	0.80

4	0.29	2.32	14.19	16.13	0.15	0.50

5	0.35	1.98	21.12	22.83	0.15	0.45

6	0.09	6.98	10.37	10.37	0.16	1.60

Mean ± SD	0.26 ± 0.10	3.19 ± 1.93	18.93 ± 10.11	21.61 ± 12.45	0.15 ± 0.08	0.64 ± 0.51

Abbreviations are the same as for Table 1.

**Figure 3:**
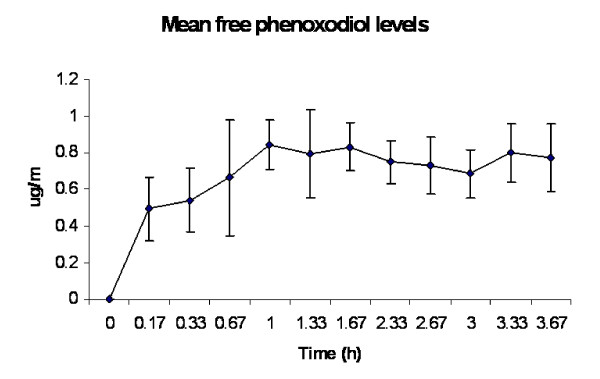
**Mean plasma concentrations of free phenoxodiol during continuous intravenous infusion of phenoxodiol at 2 mg/kg/h**.

**Figure 4:**
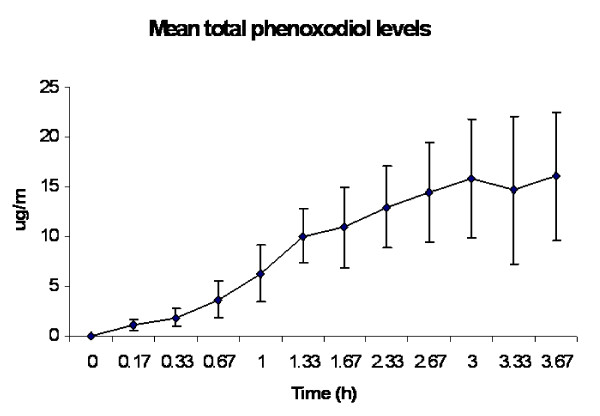
**Mean plasma concentrations of total phenoxodiol during continuous intravenous infusion of phenoxodiol at 2 mg/kg/h**.

Summaries of the pharmacokinetic data for free and total phenoxodiol from the 5 patients who had continuous intravenous dosing are presented in Tables 3 and 4. Plasma concentration versus time plots for free and total phenoxodiol for these patients are presented in Figures 3 and 4. Plasma levels of free phenoxodiol accumulated fairly rapidly with a mean accumulation half-life of 0.17 ± 0.04 hours. Plasma levels of total phenoxodiol accumulated at a relatively slower rate with a mean accumulation half life of 2.78 ± 0.96 hours. Steady state plasma levels of total phenoxodiol were more variable than steady state plasma levels of free phenoxodiol (Figures 3 and 4). The mean clearance rate for total phenoxodiol (0.06 ± 0.01 L/h) was lower than that of free phenoxodiol (1.29 ± 0.23 L/h). In two of the patients (patient 1 and patient 2) the infusion did not appear to be continued long enough for total phenoxodiol to reach steady state plasma levels. As a result, though the final plasma concentration time point in these patients was used to estimate C_ss_, accumulation half-life and clearance, the calculated values are likely to be underestimated.

**Table 3:** Pharmacokinetic parameters for free phenoxodiol infused at a dose of 2 mg/kg/h.

	Css (μg/ml)	**Time to ss (h)**	Acc T1/2 (h)	Cl (L/h)
1	0.92	0.67	0.13	1.08

2	0.65	1.00	0.20	1.53

4	0.77	1.00	0.20	1.28

5	0.66	1.00	0.20	1.51

6	0.96	0.67	0.13	1.03

Mean ± SD	0.79 ± 0.14	0.87 ± 0.18	0.17 ± 0.04	1.29 ± 0.23

Css is the plasma concentration at steady state. Time to steady state is the time taken to reach Css. Acc T1/2 is the accumulation half-life. Cl is the plasma clearance rate.

**Table 4:** Pharmacokinetic parameters for total phenoxodiol infused at a dose of 2 mg/kg/h.

Patient number	Css (μg/ml)	Time to ss (h)	Acc T1/2 (h)	Cl (L/h)
1*	14.74	3.66	0.73	0.06

2*	32.31	4.00	0.80	0.03

4	13.33	2.00	0.40	0.07

5	14.80	2.00	0.40	0.07

6	12.04	2.33	0.47	0.08

Mean ± SD	17.45 ± 8.38	2.78 ± 0.96	0.56 ± 0.19	0.06 ± 0.01

Abbreviations are the same as for table 3. * In patients 1 and 2 the infusion was not continued long enough to reach steady state and the values calculated are therefore underestimates of the valid results.

Urine excretion of free and total phenoxodiol, respectively, ranged from 0.5 mg - 5.2 mg and 13 - 42 mg after intravenous bolus dosing (data not shown). Urine excretion of phenoxodiol after continuous intravenous infusion was not measured.

### Conjugation

The degree of phenoxodiol conjugation is estimated at 91%, based on calculation of the proportion of free phenoxodiol AUC : total phenoxodiol AUC (mean 8.7%) from bolus administration. For each individual subject, estimated proportion of free phenoxodiol from the bolus data correlates very well with total phenoxodiol clearance as measured during continuous infusion (data not shown).

### Adverse events

In total, five adverse events were reported from 2 patients; no other patients had adverse events. One patient experienced 3 serious adverse events: hepatic metastasis, leg oedema and thrombocytopenia; all of these had been present prior to the study but had increased in severity during the study. They were considered to be due to disease progression and unrelated to the study medication. This patient died 7 days after continuous intravenous infusion of phenoxodiol. His death was considered to be due to rapid progression of malignant disease and unrelated to the study medication. The second patient experienced two non-serious adverse events: groin pain and nausea. The groin pain occurred the day following the single bolus dose of phenoxodiol and was considered not to be related to the study medication. The episode of nausea occurred following the continuous intravenous infusion of phenoxodiol, was mild in intensity, and was considered to be possibly related to study medication. The episode of nausea did not require intervention and resolved spontaneously.

## Discussion

This first - in - human study investigated the pharmacokinetics of phenoxodiol, a novel isoflavone, given as a single intravenous bolus injection and as a single episode of continuous intravenous infusion in the same patients. We showed that mean plasma levels of free phenoxodiol declined rapidly from an initial value of around 3 μg/ml with an apparent half-life of 0.67 h. The clearance of free phenoxodiol was relatively high (2.48 L/h) and the volume of distribution was 1.55 L/kg, indicating distribution of the drug into a body compartment approximately 2.3 times the extracellular fluid volume. Plasma levels of total phenoxodiol declined from an initial value of approximately 8 μg/ml with an apparent half-life of 3.19 h. Similar to free phenoxodiol, the log-plasma concentration plot for each patient appeared linear (data not presented) suggesting first order pharmacokinetics. The clearance of total phenoxodiol (0.15 L/h) was considerably lower than for free phenoxodiol. In contrast, the volume of distribution of total phenoxodiol (0.64 L/kg) was smaller than for free phenoxodiol. Plasma concentrations of total phenoxodiol were more variable than for free phenoxodiol. The ratio of AUC free phenoxodiol : AUC total phenoxodiol was 0.087, indicating that >90% of the drug following an intravenous bolus was present in the conjugated form. Furthermore, the initial plasma concentrations of total phenoxodiol were approximately 2.7 times the initial plasma concentrations of free phenoxodiol. These observations are most likely due to rapid conjugation of phenoxodiol in the liver and a slower clearance of conjugated than free phenoxodiol. Urine excretion of phenoxodiol was difficult to interpret because of missing data points from incomplete urine collections. Nevertheless, it appears that 8 hours was not sufficiently long for the complete collection of all phenoxodiol excreted by renal mechanisms.

Following continuous intravenous infusion, plasma concentrations of free plasma phenoxodiol rose rapidly with an apparent accumulation half-life of 0.17 h. Although this appeared to be a little shorter than the half-life estimated following the single intravenous bolus dose in Part 1 of the study, the results were also more variable in Part 1 of the study. The plasma clearance calculated during the continuous infusion (1.29 L/h) appeared a little lower than following intravenous bolus dosing, but the results were consistent with each other. Further, the degree of individual phenoxodiol conjugation estimated from the bolus part of the study correlated well with clearance measured in the continuous infusion part of the study. The mean plasma concentrations of free phenoxodiol at steady state were approximately 0.79 μg/ml, which is broadly in the range that has been demonstrated to have anti-cancer effects in *in vitro *studies.

Since this study was performed however, two publications regarding phenoxodiol pharmacokinetics have appeared in print [19,20]. In a 7 day infusion study of phenoxodiol, steady state levels were reached in 14 of 19 patients [20]. Phenoxodiol doses were escalated in patient cohorts, ranging from 0.65 mg/kg/24 h to 27 mg/kg/24 h. Overall mean accumulation half-life of conjugated phenoxodiol was 10.63 h, and time to C_ss _was 53.14 h with a clearance rate of 0.0260 L/Kg/h [20]. At the closest dose cohort to the present study (2.2 mg/kg/24 h), mean Css was 4.8 μg/ml. In a study of weekly intravenous bolus phenoxodiol [19], clearance of total phenoxodiol was found to be higher (around 82 ml/min) and half-life longer (around 300 mins) than in this study. Interestingly, clearance of phenoxodiol was similar in these two studies to our present study, but half-life was significantly longer. It is important to note that comparison across studies is problematic, since one study reported parameters concerning conjugated phenoxodiol only [20], whereas this study and the weekly bolus study measured free and total phenoxodiol. Further, the significance of conjugation is unclear, though we hypothesize that free phenoxodiol is the most biologically active, and therefore the most important moiety. Other reasons for the discrepancy between pharmacokinetic parameters in the different studies include different methods for assay (HPLC in this study, LC-MS in the other two), and the use of a different (cyclodextrin) carrier for phenoxodiol in the Phase I studies. Other than the different preparation, other possible explanations include the tight control of diet, caffeine and smoking in this study, whereas conditions were not stated in the Phase I studies.

The limitations of our study are that only a small number of patients with cancer were studied due to the fact that it was a first -in-human Phase 1 study of Phenoxodiol. In addition, we examined only a limited dose range of phenoxodiol, so we cannot comment on whether the pharmacokinetics of phenoxodiol are truly linear on the basis of this study. Furthermore, the effects of age and of renal or hepatic dysfunction on phenoxodiol pharmacokinetics are not known. Further studies are required to address these issues.

## Conclusions

In conclusion, the results of our study indicate that phenoxodiol has a very short half-life when given intravenously, particularly for the free form. Since pharmacokinetic variance within (our study) and between individuals [19,20] is very wide, it would appear that administration of phenoxodiol by continuous infusion or by chronic oral administration may be the optimal modes of administration if it considered that constant plasma levels are desirable for anti-cancer therapy.

## Competing interests

Funded by Novogen Pty Ltd, Sydney Australia. Jan B. Howes was employed by Novogen Pty Ltd at the time of the study. Paul de Souza and Laurence Howes were consultants to Novogen Pty Ltd in 2000 but were also physician investigators at St George Hospital and were involved in other studies. Leanne West and Li Huang are employees of Marshall Edwards Pty Ltd. Marshall-Edwards Pty Ltd, the owner of phenoxodiol, is a subsidiary of Novogen Pty Ltd. Novogen Pty Ltd analysed the blood samples for pharmacokinetic assessment, but did not design or conduct the study, collect or manage the data, interpret the results, or draft the manuscript. However, Novogen Pty Ltd and Marshall Edwards did have the opportunity to review and revise the manuscript.

## Authors' contributions

JBH and LGH conceived the study, wrote the study protocol, and drafted the final manuscript. LGH also analyzed the pharmacokinetic data. PDS referred patients with cancer, obtained consent, and contributed to the pharmacokinetic analysis. LW coordinated the study, and collected all the clinical trial data. LJH collected the pharmacokinetic data and performed the HPLC analyses. All authors read and approved the final version of the manuscript.

## Pre-publication history

The pre-publication history for this paper can be accessed here:

http://www.biomedcentral.com/1472-6904/11/1/prepub
